# [μ-1,4-Bis(diphenyl­phosphino­yl)butane-κ^2^ *O*:*O*′]bis­[cyclo­pentyl­diphen­yl(trifluoro­acetato-κ*O*)tin(IV)]

**DOI:** 10.1107/S1600536808013202

**Published:** 2008-05-10

**Authors:** Yin Yin Teo, Kong Mun Lo, Seik Weng Ng

**Affiliations:** a Department of Chemistry, University of Malaya, 50603 Kuala Lumpur, Malaysia

## Abstract

The mol­ecule of the dinuclear title compound, [Sn_2_(C_5_H_9_)_2_(C_6_H_5_)_4_(C_2_F_3_O_2_)_2_(C_28_H_28_O_2_P_2_)], lies on a center of inversion at the mid-point of the central C—C bond of the bridging phosphine oxide ligand. The Sn atom is five-coordinate in a *trans*-C_3_SnO_2_ trigonal-bipyramidal geometry.

## Related literature

For the 1/1 co-crystal of bis­[1,3-bis­(diphenyl­phosphino)propane]silver(I) cyclo­pentyl­diphenyl­bis(trifluoro­acetato)stannate(IV) with bis­[1,3-bis­(diphenyl­phosphino)propane]silver(I) triphenyl­bis(trifluoro­acetato)stannate(IV), see: Teo *et al.* (2004 ▶). For other oxygen-donor adducts of triorganotin carboxyl­ates, see: Ng & Kumar Das (1997 ▶). For a review of the structural chemistry of organotin carboxyl­ates, see: Tiekink (1991 ▶, 1994 ▶).
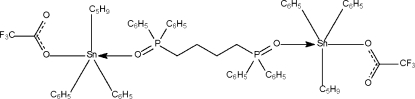


## Experimental

### 

#### Crystal data

[Sn_2_(C_5_H_9_)_2_(C_6_H_5_)_4_(C_2_F_3_O_2_)_2_(C_28_H_28_O_2_P_2_)]
*M* *_r_* = 1368.51
Monoclinic, 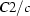

*a* = 17.3833 (3) Å
*b* = 12.9181 (2) Å
*c* = 28.2447 (4) Å
β = 110.990 (1)°
*V* = 5921.7 (2) Å^3^
*Z* = 4
Mo *K*α radiation
μ = 0.97 mm^−1^
*T* = 100 (2) K
0.25 × 0.20 × 0.15 mm

#### Data collection

Bruker SMART APEX diffractometer
Absorption correction: multi-scan (*SADABS*; Sheldrick, 1996 ▶) *T*_min_ = 0.794, *T*_max_ = 0.868
31739 measured reflections
7363 independent reflections
6394 reflections with *I* > 2σ(*I*)
*R*_int_ = 0.033

#### Refinement

*R*[*F* ^2^ > 2σ(*F* ^2^)] = 0.030
*wR*(*F* ^2^) = 0.088
*S* = 1.06
7363 reflections
370 parameters
H-atom parameters constrained
Δρ_max_ = 0.82 e Å^−3^
Δρ_min_ = −0.76 e Å^−3^

### 

Data collection: *APEX2* (Bruker, 2007 ▶); cell refinement: *SAINT* (Bruker, 2007 ▶); data reduction: *SAINT*; program(s) used to solve structure: *SHELXS97* (Sheldrick, 2008 ▶); program(s) used to refine structure: *SHELXL97* (Sheldrick, 2008 ▶); molecular graphics: *X-SEED* (Barbour, 2001 ▶); software used to prepare material for publication: *publCIF* (Westrip, 2008 ▶).

## Supplementary Material

Crystal structure: contains datablocks global, I. DOI: 10.1107/S1600536808013202/bq2075sup1.cif

Structure factors: contains datablocks I. DOI: 10.1107/S1600536808013202/bq2075Isup2.hkl

Additional supplementary materials:  crystallographic information; 3D view; checkCIF report

## Figures and Tables

**Table 1:** Selected bond angles (°)

C1—Sn1—C6	125.3 (1)
C1—Sn1—C12	120.9 (1)
C1—Sn1—O1	93.4 (1)
C1—Sn1—O3	87.5 (1)
C6—Sn1—C12	113.5 (1)
C6—Sn1—O1	93.2 (1)
C6—Sn1—O3	90.3 (1)
C12—Sn1—O1	88.2 (1)
C12—Sn1—O3	87.0 (1)
O1—Sn1—O3	174.9 (1)

**Fig. 1.**
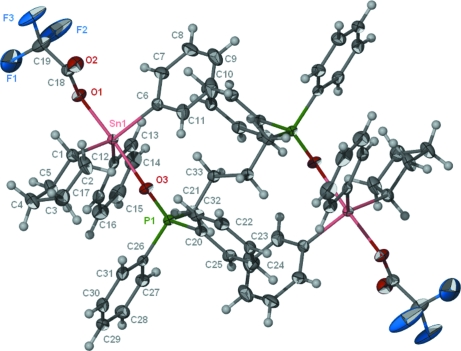
Molecular structure of (I) with the atomic numbering scheme. Displacement ellipsoids are drawn at the 70% probability level. Hydrogen atoms are drawn as spheres of arbitrary radius.

## supplementary crystallographic information

### Experimental

Triphenyltin hydroxide (0.18 g, 0.5 mmol) and chorodifluoroacetic acid (0.05 ml, 0.5 mmol) were dissolved in dichloromethane/methanol (25 ml). The mixture was heated until the hydroxide dissolved completely. Another solution containing 1,4-bis(diphenylphosphino)butane (0.42 g, 1.0 mmol) and silver trifluoroacetate (0.11 g, 0.5 mmol) was prepared; this was also heated until the reagents dissolved completely. The two solutions were mixed; crystals were obtained by allowing the solvent to evaporate.

### Refinement

Carbon-bound H-atoms were placed in calculated positions (C—H 0.95 to 0.99 Å) and were included in the refinement in the riding model approximation, with *U*(H) set to 1.2*U*_eq_(C).

### Crystal data

**Table:**

[Sn_2_(C_5_H_9_)_2_(C_6_H_5_)_4_(C_2_F_3_O_2_)_2_(C_28_H_28_O_2_P_2_)]	*F*_000_ = 2776
*M**_r_* = 1368.51	*D*_x_ = 1.535 Mg m^−^^3^
Monoclinic, *C*2/*c*	Mo *K*α radiation λ = 0.71073 Å
Hall symbol: -C 2yc	Cell parameters from 9914 reflections
*a* = 17.3833 (3) Å	θ = 2.3–28.3º
*b* = 12.9181 (2) Å	µ = 0.97 mm^−^^1^
*c* = 28.2447 (4) Å	*T* = 100 (2) K
β = 110.990 (1)º	Block, colorless
*V* = 5921.7 (2) Å^3^	0.25 × 0.20 × 0.15 mm
*Z* = 4	

### Data collection

**Table:**

Bruker SMART APEX diffractometer	7363 independent reflections
Radiation source: fine-focus sealed tube	6394 reflections with *I* > 2σ(*I*)
Monochromator: graphite	*R*_int_ = 0.033
*T* = 100(2) K	θ_max_ = 28.0º
ω scans	θ_min_ = 1.5º
Absorption correction: Multi-scan(SADABS; Sheldrick, 1996)	*h* = −23→18
*T*_min_ = 0.794, *T*_max_ = 0.868	*k* = −17→17
31739 measured reflections	*l* = −37→37

### Refinement

**Table:**

Refinement on *F*^2^	Secondary atom site location: difference Fourier map
Least-squares matrix: full	Hydrogen site location: inferred from neighbouring sites
*R*[*F*^2^ > 2σ(*F*^2^)] = 0.031	H-atom parameters constrained
*wR*(*F*^2^) = 0.088	*w* = 1/[σ^2^(*F*_o_^2^) + (0.0451*P*)^2^ + 12.9939*P*] where *P* = (*F*_o_^2^ + 2*F*_c_^2^)/3
*S* = 1.06	(Δ/σ)_max_ = 0.001
7363 reflections	Δρ_max_ = 0.82 e Å^−^^3^
370 parameters	Δρ_min_ = −0.76 e Å^−^^3^
Primary atom site location: structure-invariant direct methods	Extinction correction: none

### Fractional atomic coordinates and isotropic or equivalent isotropic displacement parameters (Å^2^)

**Table:**

	*x*	*y*	*z*	*U*_iso_*/*U*_eq_	
Sn1	0.407012 (10)	0.526354 (11)	0.127309 (6)	0.01344 (6)	
P1	0.42844 (4)	0.77398 (4)	0.07214 (2)	0.01343 (12)	
F1	0.4117 (2)	0.2664 (2)	0.25350 (8)	0.0863 (11)	
F2	0.29101 (16)	0.25091 (18)	0.20104 (13)	0.0759 (9)	
F3	0.38056 (15)	0.14285 (14)	0.19985 (9)	0.0524 (6)	
O1	0.37049 (12)	0.40659 (13)	0.17112 (7)	0.0196 (4)	
O2	0.42139 (13)	0.27301 (15)	0.14034 (7)	0.0259 (4)	
O3	0.44361 (11)	0.66128 (13)	0.08677 (7)	0.0177 (4)	
C1	0.53511 (16)	0.49164 (19)	0.16173 (10)	0.0189 (5)	
H1	0.5396	0.4217	0.1777	0.023*	
C2	0.58724 (17)	0.4890 (2)	0.12735 (10)	0.0266 (6)	
H2A	0.5945	0.4168	0.1179	0.032*	
H2B	0.5599	0.5292	0.0960	0.032*	
C3	0.67078 (19)	0.5373 (2)	0.15837 (11)	0.0270 (6)	
H3A	0.6768	0.6060	0.1446	0.032*	
H3B	0.7165	0.4922	0.1578	0.032*	
C4	0.67096 (18)	0.5474 (2)	0.21267 (11)	0.0265 (6)	
H4A	0.6904	0.4828	0.2322	0.032*	
H4B	0.7062	0.6057	0.2309	0.032*	
C5	0.58063 (17)	0.5679 (2)	0.20397 (10)	0.0258 (6)	
H5A	0.5654	0.6403	0.1931	0.031*	
H5B	0.5685	0.5540	0.2351	0.031*	
C6	0.32668 (16)	0.46764 (18)	0.05610 (10)	0.0177 (5)	
C7	0.25672 (17)	0.4121 (2)	0.05365 (11)	0.0252 (6)	
H7	0.2454	0.4012	0.0838	0.030*	
C8	0.2032 (2)	0.3724 (2)	0.00810 (13)	0.0361 (8)	
H8	0.1556	0.3351	0.0072	0.043*	
C9	0.2192 (2)	0.3870 (2)	−0.03592 (12)	0.0386 (8)	
H9	0.1828	0.3598	−0.0672	0.046*	
C10	0.2891 (2)	0.4418 (2)	−0.03419 (11)	0.0341 (7)	
H10	0.3008	0.4512	−0.0643	0.041*	
C11	0.34163 (18)	0.4828 (2)	0.01122 (10)	0.0233 (6)	
H11	0.3884	0.5217	0.0118	0.028*	
C12	0.35061 (16)	0.63869 (18)	0.15955 (9)	0.0157 (5)	
C13	0.26575 (17)	0.6385 (2)	0.14839 (10)	0.0217 (5)	
H13	0.2331	0.5857	0.1272	0.026*	
C14	0.22813 (19)	0.7136 (2)	0.16749 (11)	0.0282 (6)	
H14	0.1701	0.7127	0.1590	0.034*	
C15	0.2752 (2)	0.7901 (2)	0.19905 (11)	0.0301 (6)	
H15	0.2495	0.8410	0.2126	0.036*	
C16	0.3596 (2)	0.7924 (2)	0.21080 (11)	0.0281 (6)	
H16	0.3917	0.8450	0.2324	0.034*	
C17	0.39753 (17)	0.71774 (19)	0.19097 (9)	0.0199 (5)	
H17	0.4554	0.7203	0.1987	0.024*	
C18	0.38999 (16)	0.31217 (18)	0.16788 (10)	0.0182 (5)	
C19	0.36869 (19)	0.2422 (2)	0.20599 (12)	0.0284 (6)	
C20	0.45092 (15)	0.79534 (18)	0.01553 (9)	0.0147 (5)	
C21	0.49076 (16)	0.71801 (18)	−0.00151 (10)	0.0177 (5)	
H21	0.5054	0.6547	0.0167	0.021*	
C22	0.50906 (16)	0.73336 (19)	−0.04496 (10)	0.0196 (5)	
H22	0.5364	0.6808	−0.0564	0.023*	
C23	0.48729 (16)	0.8256 (2)	−0.07160 (9)	0.0192 (5)	
H23	0.4991	0.8356	−0.1016	0.023*	
C24	0.44851 (16)	0.90303 (19)	−0.05467 (10)	0.0190 (5)	
H24	0.4341	0.9662	−0.0730	0.023*	
C25	0.43071 (16)	0.88863 (18)	−0.01101 (10)	0.0175 (5)	
H25	0.4048	0.9422	0.0008	0.021*	
C26	0.49735 (15)	0.85895 (18)	0.11890 (9)	0.0155 (5)	
C27	0.47046 (17)	0.94103 (19)	0.14122 (10)	0.0196 (5)	
H27	0.4131	0.9529	0.1326	0.024*	
C28	0.52691 (18)	1.0053 (2)	0.17594 (10)	0.0224 (5)	
H28	0.5082	1.0607	0.1912	0.027*	
C29	0.61071 (18)	0.9889 (2)	0.18842 (10)	0.0228 (5)	
H29	0.6492	1.0333	0.2120	0.027*	
C30	0.63854 (17)	0.9076 (2)	0.16660 (10)	0.0230 (5)	
H30	0.6960	0.8964	0.1752	0.028*	
C31	0.58197 (16)	0.84317 (19)	0.13223 (10)	0.0192 (5)	
H31	0.6010	0.7873	0.1175	0.023*	
C32	0.32534 (15)	0.81759 (18)	0.06190 (9)	0.0159 (5)	
H32A	0.3193	0.8242	0.0953	0.019*	
H32B	0.3185	0.8876	0.0467	0.019*	
C33	0.25520 (15)	0.74897 (19)	0.02804 (9)	0.0168 (5)	
H33A	0.2656	0.6767	0.0404	0.020*	
H33B	0.2030	0.7717	0.0314	0.020*	

### Atomic displacement parameters (Å^2^)

**Table:**

	*U*^11^	*U*^22^	*U*^33^	*U*^12^	*U*^13^	*U*^23^
Sn1	0.01497 (9)	0.01195 (8)	0.01275 (9)	−0.00079 (6)	0.00417 (6)	−0.00029 (5)
P1	0.0143 (3)	0.0118 (3)	0.0149 (3)	−0.0004 (2)	0.0061 (2)	0.0006 (2)
F1	0.149 (3)	0.0775 (18)	0.0259 (11)	−0.0438 (19)	0.0234 (15)	0.0105 (11)
F2	0.0637 (16)	0.0447 (12)	0.156 (3)	0.0222 (11)	0.0836 (19)	0.0462 (15)
F3	0.0724 (16)	0.0185 (8)	0.0899 (17)	0.0124 (9)	0.0578 (14)	0.0185 (9)
O1	0.0267 (10)	0.0141 (8)	0.0214 (9)	0.0011 (7)	0.0125 (8)	0.0015 (7)
O2	0.0327 (11)	0.0236 (9)	0.0254 (10)	0.0019 (8)	0.0153 (9)	−0.0021 (8)
O3	0.0208 (9)	0.0128 (8)	0.0224 (9)	−0.0001 (7)	0.0111 (7)	0.0025 (6)
C1	0.0170 (12)	0.0187 (11)	0.0188 (12)	0.0009 (9)	0.0036 (10)	−0.0009 (9)
C2	0.0212 (14)	0.0369 (15)	0.0197 (13)	0.0039 (12)	0.0048 (11)	−0.0061 (11)
C3	0.0235 (14)	0.0299 (14)	0.0277 (15)	0.0019 (11)	0.0093 (12)	0.0043 (11)
C4	0.0207 (14)	0.0297 (13)	0.0235 (14)	0.0006 (11)	0.0010 (11)	−0.0058 (11)
C5	0.0211 (14)	0.0346 (14)	0.0177 (13)	0.0027 (11)	0.0020 (11)	−0.0072 (11)
C6	0.0173 (12)	0.0146 (10)	0.0175 (12)	0.0027 (9)	0.0016 (10)	−0.0005 (9)
C7	0.0218 (14)	0.0181 (12)	0.0291 (14)	−0.0029 (10)	0.0009 (11)	−0.0001 (10)
C8	0.0259 (16)	0.0207 (13)	0.0439 (19)	−0.0029 (11)	−0.0093 (14)	−0.0035 (12)
C9	0.043 (2)	0.0209 (13)	0.0313 (16)	0.0073 (13)	−0.0116 (14)	−0.0109 (12)
C10	0.045 (2)	0.0332 (15)	0.0172 (13)	0.0154 (14)	0.0030 (13)	−0.0053 (11)
C11	0.0262 (15)	0.0225 (12)	0.0184 (13)	0.0062 (10)	0.0045 (11)	−0.0014 (10)
C12	0.0222 (13)	0.0156 (10)	0.0101 (10)	0.0022 (9)	0.0066 (9)	0.0021 (8)
C13	0.0225 (13)	0.0208 (12)	0.0220 (13)	−0.0006 (10)	0.0080 (11)	0.0018 (10)
C14	0.0271 (15)	0.0296 (14)	0.0325 (15)	0.0073 (12)	0.0162 (13)	0.0042 (12)
C15	0.0444 (18)	0.0237 (13)	0.0299 (15)	0.0120 (12)	0.0227 (14)	0.0038 (11)
C16	0.0421 (18)	0.0210 (12)	0.0238 (14)	0.0002 (12)	0.0147 (13)	−0.0052 (10)
C17	0.0251 (14)	0.0188 (11)	0.0146 (11)	0.0002 (10)	0.0055 (10)	0.0010 (9)
C18	0.0166 (12)	0.0169 (11)	0.0209 (12)	−0.0002 (9)	0.0064 (10)	−0.0003 (9)
C19	0.0332 (16)	0.0185 (12)	0.0400 (17)	0.0037 (11)	0.0210 (14)	0.0064 (11)
C20	0.0135 (11)	0.0144 (10)	0.0159 (11)	−0.0025 (8)	0.0049 (9)	−0.0006 (8)
C21	0.0191 (12)	0.0139 (10)	0.0214 (12)	−0.0003 (9)	0.0087 (10)	0.0013 (9)
C22	0.0189 (13)	0.0179 (11)	0.0232 (13)	−0.0007 (9)	0.0091 (10)	−0.0031 (9)
C23	0.0193 (13)	0.0247 (12)	0.0137 (11)	−0.0033 (10)	0.0060 (10)	0.0005 (9)
C24	0.0199 (13)	0.0168 (11)	0.0190 (12)	−0.0001 (9)	0.0054 (10)	0.0044 (9)
C25	0.0174 (12)	0.0143 (10)	0.0214 (12)	0.0008 (9)	0.0077 (10)	0.0008 (9)
C26	0.0157 (12)	0.0138 (10)	0.0160 (11)	−0.0013 (9)	0.0045 (9)	0.0035 (8)
C27	0.0179 (13)	0.0189 (11)	0.0218 (13)	−0.0012 (9)	0.0068 (10)	−0.0009 (9)
C28	0.0252 (14)	0.0203 (11)	0.0218 (13)	−0.0021 (10)	0.0084 (11)	−0.0037 (10)
C29	0.0235 (14)	0.0206 (12)	0.0199 (13)	−0.0063 (10)	0.0025 (11)	0.0013 (10)
C30	0.0162 (13)	0.0250 (13)	0.0230 (13)	0.0003 (10)	0.0013 (11)	0.0046 (10)
C31	0.0171 (12)	0.0175 (11)	0.0215 (12)	0.0036 (9)	0.0052 (10)	0.0036 (9)
C32	0.0147 (12)	0.0179 (11)	0.0170 (11)	0.0003 (9)	0.0080 (9)	−0.0005 (9)
C33	0.0149 (12)	0.0183 (11)	0.0174 (12)	−0.0015 (9)	0.0059 (10)	0.0011 (9)

### Geometric parameters (Å, °)

**Table:**

Sn1—C1	2.134 (3)	C12—C17	1.406 (3)
Sn1—C12	2.130 (2)	C13—C14	1.382 (4)
Sn1—C6	2.135 (2)	C13—H13	0.9500
Sn1—O1	2.212 (2)	C14—C15	1.385 (4)
Sn1—O3	2.297 (2)	C14—H14	0.9500
P1—O3	1.5105 (17)	C15—C16	1.383 (5)
P1—C20	1.798 (2)	C15—H15	0.9500
P1—C32	1.801 (3)	C16—C17	1.394 (4)
P1—C26	1.803 (2)	C16—H16	0.9500
F1—C19	1.318 (4)	C17—H17	0.9500
F2—C19	1.312 (4)	C18—C19	1.549 (4)
F3—C19	1.321 (3)	C20—C21	1.396 (3)
O1—C18	1.278 (3)	C20—C25	1.396 (3)
O2—C18	1.210 (3)	C21—C22	1.388 (4)
C1—C5	1.531 (4)	C21—H21	0.9500
C1—C2	1.548 (4)	C22—C23	1.387 (4)
C1—H1	1.0000	C22—H22	0.9500
C2—C3	1.534 (4)	C23—C24	1.384 (4)
C2—H2A	0.9900	C23—H23	0.9500
C2—H2B	0.9900	C24—C25	1.387 (4)
C3—C4	1.538 (4)	C24—H24	0.9500
C3—H3A	0.9900	C25—H25	0.9500
C3—H3B	0.9900	C26—C31	1.397 (3)
C4—C5	1.523 (4)	C26—C27	1.397 (3)
C4—H4A	0.9900	C27—C28	1.388 (4)
C4—H4B	0.9900	C27—H27	0.9500
C5—H5A	0.9900	C28—C29	1.387 (4)
C5—H5B	0.9900	C28—H28	0.9500
C6—C7	1.392 (4)	C29—C30	1.389 (4)
C6—C11	1.396 (4)	C29—H29	0.9500
C7—C8	1.388 (4)	C30—C31	1.385 (4)
C7—H7	0.9500	C30—H30	0.9500
C8—C9	1.381 (5)	C31—H31	0.9500
C8—H8	0.9500	C32—C33	1.534 (3)
C9—C10	1.392 (5)	C32—H32A	0.9900
C9—H9	0.9500	C32—H32B	0.9900
C10—C11	1.386 (4)	C33—C33^i^	1.529 (5)
C10—H10	0.9500	C33—H33A	0.9900
C11—H11	0.9500	C33—H33B	0.9900
C12—C13	1.394 (4)		
			
C1—Sn1—C6	125.3 (1)	C12—C13—H13	119.3
C1—Sn1—C12	120.9 (1)	C13—C14—C15	119.9 (3)
C1—Sn1—O1	93.4 (1)	C13—C14—H14	120.0
C1—Sn1—O3	87.5 (1)	C15—C14—H14	120.0
C6—Sn1—C12	113.5 (1)	C14—C15—C16	120.0 (3)
C6—Sn1—O1	93.2 (1)	C14—C15—H15	120.0
C6—Sn1—O3	90.3 (1)	C16—C15—H15	120.0
C12—Sn1—O1	88.2 (1)	C15—C16—C17	120.2 (3)
C12—Sn1—O3	87.0 (1)	C15—C16—H16	119.9
O1—Sn1—O3	174.9 (1)	C17—C16—H16	119.9
O3—P1—C20	108.59 (11)	C16—C17—C12	120.3 (3)
O3—P1—C32	114.31 (11)	C16—C17—H17	119.8
C20—P1—C32	109.27 (11)	C12—C17—H17	119.8
O3—P1—C26	112.42 (11)	O2—C18—O1	129.6 (2)
C20—P1—C26	105.01 (11)	O2—C18—C19	118.6 (2)
C32—P1—C26	106.80 (11)	O1—C18—C19	111.8 (2)
C18—O1—Sn1	119.40 (16)	F2—C19—F3	105.8 (3)
P1—O3—Sn1	144.62 (11)	F2—C19—F1	105.9 (3)
C5—C1—C2	104.4 (2)	F3—C19—F1	107.8 (3)
C5—C1—Sn1	112.55 (17)	F2—C19—C18	111.7 (2)
C2—C1—Sn1	117.95 (18)	F3—C19—C18	112.8 (2)
C5—C1—H1	107.1	F1—C19—C18	112.3 (2)
C2—C1—H1	107.1	C21—C20—C25	119.6 (2)
Sn1—C1—H1	107.1	C21—C20—P1	119.31 (18)
C3—C2—C1	106.2 (2)	C25—C20—P1	121.12 (19)
C3—C2—H2A	110.5	C22—C21—C20	120.1 (2)
C1—C2—H2A	110.5	C22—C21—H21	119.9
C3—C2—H2B	110.5	C20—C21—H21	119.9
C1—C2—H2B	110.5	C21—C22—C23	119.9 (2)
H2A—C2—H2B	108.7	C21—C22—H22	120.1
C2—C3—C4	105.5 (2)	C23—C22—H22	120.1
C2—C3—H3A	110.6	C24—C23—C22	120.3 (2)
C4—C3—H3A	110.6	C24—C23—H23	119.8
C2—C3—H3B	110.6	C22—C23—H23	119.8
C4—C3—H3B	110.6	C23—C24—C25	120.1 (2)
H3A—C3—H3B	108.8	C23—C24—H24	119.9
C5—C4—C3	102.7 (2)	C25—C24—H24	119.9
C5—C4—H4A	111.2	C24—C25—C20	120.0 (2)
C3—C4—H4A	111.2	C24—C25—H25	120.0
C5—C4—H4B	111.2	C20—C25—H25	120.0
C3—C4—H4B	111.2	C31—C26—C27	118.7 (2)
H4A—C4—H4B	109.1	C31—C26—P1	117.90 (19)
C4—C5—C1	103.3 (2)	C27—C26—P1	123.42 (19)
C4—C5—H5A	111.1	C28—C27—C26	120.5 (2)
C1—C5—H5A	111.1	C28—C27—H27	119.8
C4—C5—H5B	111.1	C26—C27—H27	119.8
C1—C5—H5B	111.1	C27—C28—C29	120.0 (3)
H5A—C5—H5B	109.1	C27—C28—H28	120.0
C7—C6—C11	118.1 (2)	C29—C28—H28	120.0
C7—C6—Sn1	119.7 (2)	C28—C29—C30	120.3 (2)
C11—C6—Sn1	122.3 (2)	C28—C29—H29	119.9
C8—C7—C6	121.3 (3)	C30—C29—H29	119.9
C8—C7—H7	119.3	C31—C30—C29	119.5 (3)
C6—C7—H7	119.3	C31—C30—H30	120.3
C9—C8—C7	120.0 (3)	C29—C30—H30	120.3
C9—C8—H8	120.0	C30—C31—C26	121.1 (2)
C7—C8—H8	120.0	C30—C31—H31	119.5
C8—C9—C10	119.5 (3)	C26—C31—H31	119.5
C8—C9—H9	120.2	C33—C32—P1	116.27 (17)
C10—C9—H9	120.2	C33—C32—H32A	108.2
C11—C10—C9	120.3 (3)	P1—C32—H32A	108.2
C11—C10—H10	119.8	C33—C32—H32B	108.2
C9—C10—H10	119.8	P1—C32—H32B	108.2
C10—C11—C6	120.7 (3)	H32A—C32—H32B	107.4
C10—C11—H11	119.6	C33^i^—C33—C32	114.1 (3)
C6—C11—H11	119.6	C33^i^—C33—H33A	108.7
C13—C12—C17	118.1 (2)	C32—C33—H33A	108.7
C13—C12—Sn1	120.94 (18)	C33^i^—C33—H33B	108.7
C17—C12—Sn1	120.86 (19)	C32—C33—H33B	108.7
C14—C13—C12	121.4 (3)	H33A—C33—H33B	107.6
C14—C13—H13	119.3		
			
C12—Sn1—O1—C18	−179.10 (19)	C17—C12—C13—C14	0.0 (4)
C1—Sn1—O1—C18	−58.3 (2)	Sn1—C12—C13—C14	−177.4 (2)
C6—Sn1—O1—C18	67.4 (2)	C12—C13—C14—C15	−1.0 (4)
C20—P1—O3—Sn1	−152.55 (17)	C13—C14—C15—C16	1.0 (4)
C32—P1—O3—Sn1	−30.3 (2)	C14—C15—C16—C17	−0.1 (4)
C26—P1—O3—Sn1	91.7 (2)	C15—C16—C17—C12	−1.0 (4)
C12—Sn1—O3—P1	−14.6 (2)	C13—C12—C17—C16	1.0 (4)
C1—Sn1—O3—P1	−135.7 (2)	Sn1—C12—C17—C16	178.35 (19)
C6—Sn1—O3—P1	98.9 (2)	Sn1—O1—C18—O2	−6.8 (4)
C12—Sn1—C1—C5	−10.2 (2)	Sn1—O1—C18—C19	173.03 (17)
C6—Sn1—C1—C5	163.39 (18)	O2—C18—C19—F2	−125.6 (3)
O1—Sn1—C1—C5	−100.18 (19)	O1—C18—C19—F2	54.5 (3)
O3—Sn1—C1—C5	74.84 (19)	O2—C18—C19—F3	−6.6 (4)
C12—Sn1—C1—C2	−131.82 (19)	O1—C18—C19—F3	173.6 (3)
C6—Sn1—C1—C2	41.8 (2)	O2—C18—C19—F1	115.5 (3)
O1—Sn1—C1—C2	138.19 (19)	O1—C18—C19—F1	−64.3 (3)
O3—Sn1—C1—C2	−46.78 (19)	O3—P1—C20—C21	−11.0 (2)
C5—C1—C2—C3	15.7 (3)	C32—P1—C20—C21	−136.3 (2)
Sn1—C1—C2—C3	141.46 (19)	C26—P1—C20—C21	109.4 (2)
C1—C2—C3—C4	10.5 (3)	O3—P1—C20—C25	170.3 (2)
C2—C3—C4—C5	−32.7 (3)	C32—P1—C20—C25	45.0 (2)
C3—C4—C5—C1	42.7 (3)	C26—P1—C20—C25	−69.3 (2)
C2—C1—C5—C4	−36.2 (3)	C25—C20—C21—C22	−0.9 (4)
Sn1—C1—C5—C4	−165.27 (18)	P1—C20—C21—C22	−179.61 (19)
C12—Sn1—C6—C7	−63.7 (2)	C20—C21—C22—C23	−0.3 (4)
C1—Sn1—C6—C7	122.3 (2)	C21—C22—C23—C24	1.0 (4)
O1—Sn1—C6—C7	25.7 (2)	C22—C23—C24—C25	−0.4 (4)
O3—Sn1—C6—C7	−150.6 (2)	C23—C24—C25—C20	−0.9 (4)
C12—Sn1—C6—C11	116.3 (2)	C21—C20—C25—C24	1.5 (4)
C1—Sn1—C6—C11	−57.7 (2)	P1—C20—C25—C24	−179.82 (19)
O1—Sn1—C6—C11	−154.2 (2)	O3—P1—C26—C31	56.0 (2)
O3—Sn1—C6—C11	29.4 (2)	C20—P1—C26—C31	−61.9 (2)
C11—C6—C7—C8	0.2 (4)	C32—P1—C26—C31	−177.84 (19)
Sn1—C6—C7—C8	−179.7 (2)	O3—P1—C26—C27	−125.9 (2)
C6—C7—C8—C9	0.4 (4)	C20—P1—C26—C27	116.2 (2)
C7—C8—C9—C10	−0.1 (4)	C32—P1—C26—C27	0.3 (2)
C8—C9—C10—C11	−1.0 (4)	C31—C26—C27—C28	−0.1 (4)
C9—C10—C11—C6	1.7 (4)	P1—C26—C27—C28	−178.2 (2)
C7—C6—C11—C10	−1.3 (4)	C26—C27—C28—C29	0.5 (4)
Sn1—C6—C11—C10	178.7 (2)	C27—C28—C29—C30	−0.5 (4)
C1—Sn1—C12—C13	−158.74 (19)	C28—C29—C30—C31	0.0 (4)
C6—Sn1—C12—C13	27.0 (2)	C29—C30—C31—C26	0.4 (4)
O1—Sn1—C12—C13	−65.8 (2)	C27—C26—C31—C30	−0.4 (4)
O3—Sn1—C12—C13	116.0 (2)	P1—C26—C31—C30	177.8 (2)
C1—Sn1—C12—C17	24.0 (2)	O3—P1—C32—C33	−48.0 (2)
C6—Sn1—C12—C17	−150.30 (18)	C20—P1—C32—C33	73.9 (2)
O1—Sn1—C12—C17	116.93 (19)	C26—P1—C32—C33	−173.00 (17)
O3—Sn1—C12—C17	−61.31 (19)	P1—C32—C33—C33^i^	−70.9 (3)

Symmetry codes: (i) −*x*+1/2, −*y*+3/2, −*z*.
